# Weapon injuries in the crusader mass graves from a 13th century attack on the port city of Sidon (Lebanon)

**DOI:** 10.1371/journal.pone.0256517

**Published:** 2021-08-25

**Authors:** Richard N. R. Mikulski, Holger Schutkowski, Martin J. Smith, Claude Doumet-Serhal, Piers D. Mitchell

**Affiliations:** 1 Department of Archaeology and Anthropology, Faculty of Science & Technology, Bournemouth University, Poole, Dorset, United Kingdom; 2 Sidon Excavations, Saida, Lebanon; 3 Department of Archaeology, University of Cambridge, Cambridge, United Kingdom; University of Otago, NEW ZEALAND

## Abstract

Archaeological excavations close to St Louis’ castle in Sidon, Lebanon have revealed two mass grave deposits containing partially articulated and disarticulated human skeletal remains. A minimum of 25 male individuals have been recovered, with no females or young children. Radiocarbon dating of the human remains, a crusader coin, and the design of Frankish belt buckles strongly indicate they belong to a single event in the mid-13th century CE. The skeletal remains demonstrate a high prevalence of unhealed sharp force, penetrating force and blunt force trauma consistent with medieval weaponry. Higher numbers of wounds on the back of individuals than the front suggests some were attacked from behind, possibly as they fled. The concentration of blade wounds to the back of the neck of others would be compatible with execution by decapitation following their capture. Taphonomic changes indicate the skeletal remains were left exposed for some weeks prior to being collected together and re-deposited in the defensive ditch by a fortified gateway within the town wall. Charring on some bones provides evidence of burning of the bodies. The findings imply the systematic clearance of partially decomposed corpses following an attack on the city, where adult and teenage males died as a result of weapon related trauma. The skeletons date from the second half of the Crusader period, when Christian-held Sidon came under direct assault from both the Mamluk Sultanate (1253 CE) and the Ilkhanate Mongols (1260 CE). It is likely that those in the mass graves died during one of these assaults.

## Introduction

The crusades to the medieval eastern Mediterranean (1097–1291 CE) saw a dramatic confluence of Eastern and Western societies in the Levant. Following a call for military aid from Europe by the Byzantine emperor, Alexius I Comnenus, the first crusade from 1097–1099 resulted in the establishment of European rule over the coastal parts of what is now Syria, Lebanon, Israel, Palestine and Jordan. From the late 12^th^ century, in the aftermath of the disastrous battle of Hattin in 1187, the Frankish states in the Latin East are generally considered to have been on the defensive, whilst beset by internal divisions which undermined their security still further [1, 2].

Knowledge of the crusaders, settlers and their lives is mainly derived from historical texts. Life in the Latin East appears to have been one where the risk of injury was widespread, not simply as a result of open warfare but also from natural disasters such as earthquakes, accidents at construction projects, crime, assassination, internecine fighting, torture, and more mundane daily risks such as those associated with horse-riding [3, 4].

Study of human remains from the crusader period cemeteries at the coastal city of Caesarea has demonstrated a range of skeletal trauma in the population there, but no evidence for weapon injuries [5]. However, several fortified crusader sites have yielded archaeological evidence of warfare [6–8]. To date, only a single context has been found where crusader period human remains present clear evidence of perimortem injuries directly related to military action. Five male individuals were recovered from a layer of ash underneath a collapsed building at the border castle of Vadum Iacob in the Kingdom of Jerusalem. The skeletal remains showed evidence for blade wounds, limb amputation, and embedded arrow heads. Historical accounts record that this castle had been attacked and destroyed by an army led by Saladin in 1179 CE [9]. For the Late medieval period in general, only a very few conflict-related mass grave sites in Europe or the Middle East have been identified, and the majority of these date to the latter centuries of the period [10–13].

The aim of this study is to improve our knowledge of warfare during the crusades through the detailed analysis of skeletal remains recovered from mass burials in the dry moat of the crusader castle at Sidon. We determine the date the event occurred, the demographic composition of the group of excavated individuals, their weapon injuries, and the manner in which survivors later dealt with the decomposing bodies.

### Medieval Sidon

The port city of Sidon is situated on the eastern Mediterranean coast of south Lebanon, located south of Beirut and north of Tyre. The surrounding geographic landscape constitutes a coastal plain broadly defined by two rivers: the Nahr el-Līṭānī to the south and the Nahr el-Awalī to the north, with the plain bordered by the foothills of the Mount Lebanon range to the east [14]. With its origins dating back to the Early Bronze Age period, Sidon was already a substantial urban settlement by the time of the crusades to the Levant (1097–1291 CE). Once under Christian control, the city and its harbour became a key strategic port in the string of coastal settlements which formed the backbone of the Frankish States in the Latin East, particularly during the 13^th^ century CE. During this period the city changed hands several times between Muslim and Frankish control [15, 16], and suffered several violent events of note during this time as reported in contemporary accounts [17–22].

## Materials and methods

**Fig 1:**
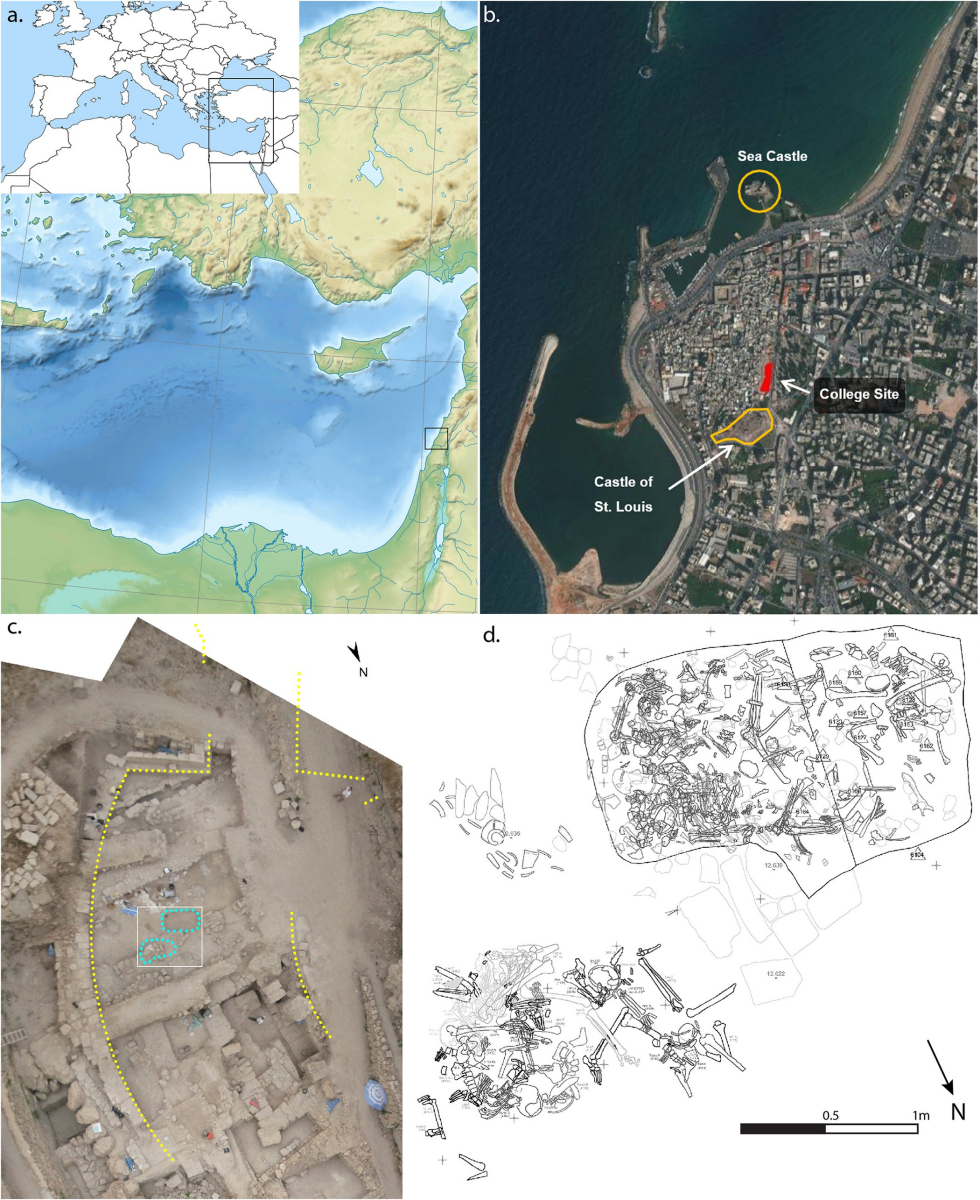
**Site location and context**: a) Location of Sidon; b) Satellite image of Sidon showing site and other key features (Sources: Esri, DigitalGlobe, GeoEye, Earthstar Geographics, CNES/Airbus DS, USDA, USGS, AeroGRID, IGN, and the GIS User Community, [26]); c) Rectified aerial photograph of context of burials 101 and 110 (blue) showing their locations and associated features; d) Digitised drawings of burials 101 and 110 (images: a) M.J. Smith; c) & d) courtesy of C. Doumet-Serhal/Directorate General of Antiquities, Lebanon (DGA)). Examples of metal artefacts recovered with the skeletal remains are provided in S1 Fig.

**Fig 2:**
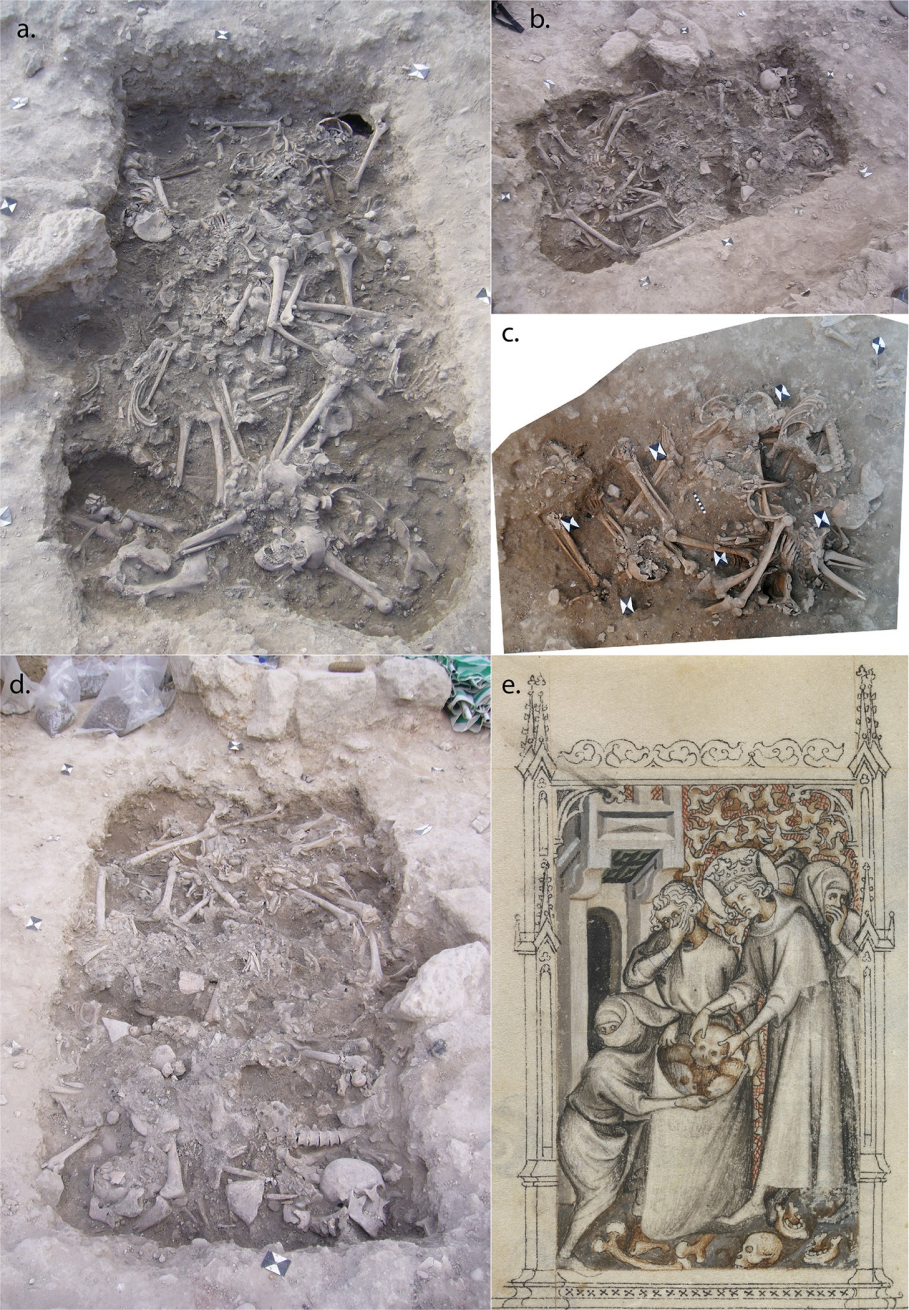
**Images of the mass graves at Sidon**: a) Burial 110, layer 8, looking east; b) Burial 110, showing how the rectangular grave has truncated one of the earlier Iron Age stone post pads; c) Rectified photograph of burial 101, demonstrating the greater degree of skeletal articulation; d) Burial 110, layer 4, looking west; e) Detail of Jean Pucelle’s *The Hours of Jeanne D’Evreux*, depicting King Louis IX of France helping to collect and bury the remains of those killed in the Mamluk raid on Sidon in 1253 CE (images: a), b) & d) R. Mikulski/DGA; c) courtesy of C. Doumet-Serhal/DGA; e) The Metropolitan Museum of Art, New York, The Cloisters Collection 1954, MS 54.1.2, fol. 159v, www.metmuseum.org).

The main excavation area, known as College Site, lies to the north-northeast of the castle of St. Louis (Qalat al-Muizz), running along part of the landward (eastern) edge of the old town district (Fig 1A–1D). The human remains derive from two deposits excavated within the medieval town fortification ditch of the city (Fig 1B and 1C). Subsequent excavation in the immediate area of the deposits has demonstrated the presence of a fortified gateway in the town wall and a bridging area over the ditch, just south of the mass grave deposits [23]. The mass grave contexts constituted a rectilinear grave pit and its fill, cut into the floor of the medieval ditch (known as burial 110, Fig 2A, 2B and 2D), and a surface deposit of human skeletal remains (burial 101, Fig 2C) situated approximately half a metre to the northeast, just outside the grave. The two deposits consisted of dense concentrations of partially articulated and disarticulated human skeletal remains, interspersed with occasional faunal remains and associated artefacts (Fig 1D; S1 Fig).

The composition of the assemblage, the taphonomic evidence, and the stratigraphy suggests an extended post-mortem interval prior to final deposition within the fortification ditch. The rectilinear grave cut contained comparatively more disarticulated human material by volume and/or smaller articulated portions of individuals, whilst the smaller assemblage which lay just outside the pit, included larger articulated sections. Two sacral fragments were matched between burials 101 and 110, indicating that the two deposits were highly likely to have been derived from the same event [24, 25]. Taphonomic observations indicated the presence of several fires amongst the material within the rectilinear grave cut, along with well-defined charring indicating limited burning to some individual bones within the assemblage lying outside the pit (burial 101), although there was no evidence of *in situ* burning associated with this latter group.

Within the grave pit (burial 110) a wide variety of artefacts were observed dispersed amongst the human and non-human bones, with no immediate patterning evident. Metal finds included copper alloy buckles and fittings, at least two different sizes of iron nails, other iron fittings, a silver coin, a silver finger-ring and a single copper alloy arrowhead (S1 Fig). Other finds included medieval potsherds, residual Persian period potsherds, glass fragments, and a small piece of charred, twisted fibre. Excepting the arrowhead, no direct physical evidence of any weapons was recovered from either inside or outside the grave pit. The copper alloy buckles are consistent with a Frankish type [27, 28], belonging to a broad timeframe spanning the early to late medieval periods. The iron nails are consistent with other finds of such artefacts from similar Crusader period contexts such as Burj Al-Ahmar [29]. The single coin, a silver denier, dates to the mid-13th century, providing a *terminus postquem* of 1245 CE [30].

Determinations of biological sex were based on assessment of sexually dimorphic traits of the ossa coxae, crania and mandibulae of adult human bones or fragments thereof [31, 32]. Estimated age at death followed standard assessment of age-related changes in the os pubis and auricular surface [33–35], in addition to the stage of fusion of skeletal elements in immature individuals [36].

**Fig 3:**
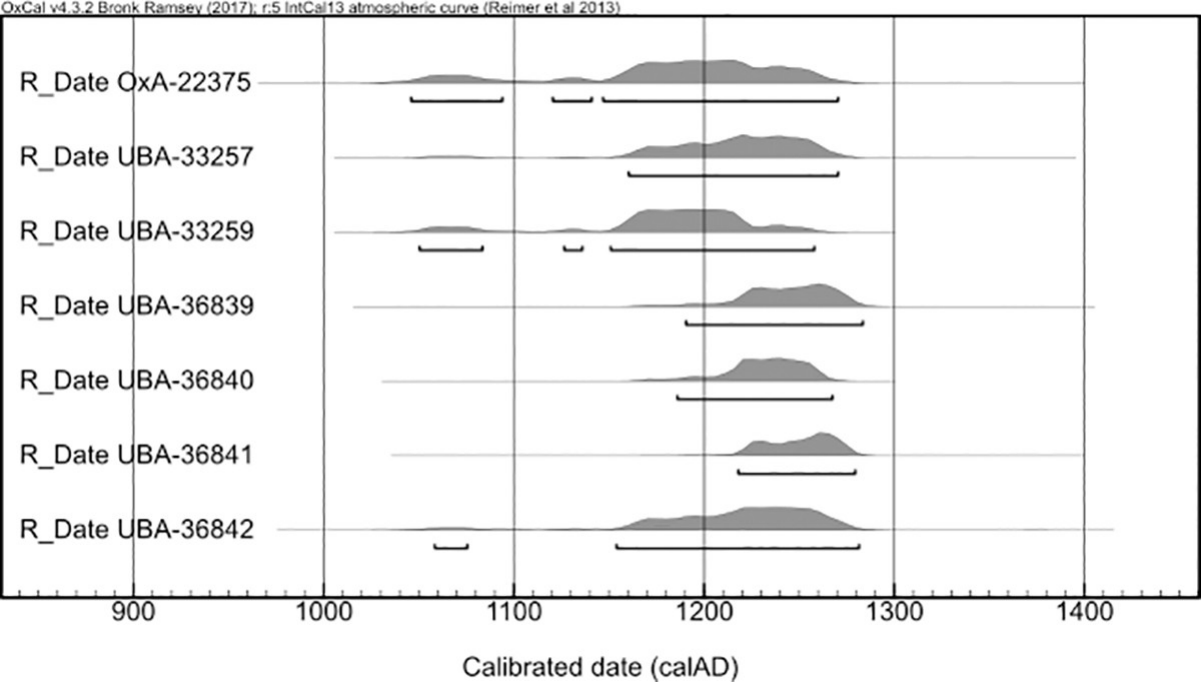
Calibrated age probability distributions of human skeletal radiocarbon samples from burials 101 and 110, excluding outliers.

A ^1^⁴C programme was undertaken in order to investigate the chronological age of the human remains and to support efforts to identify whether the two deposits were contemporary or if they represented two separate periods of deposition (Fig 3). Mandibles from two individuals were also sampled twice, testing the first and third permanent molars in each case, in an attempt to refine the potential date-range for these individuals, following Millard [37]. A T-test demonstrates that the vast majority of samples clustered around the early to mid-13^th^ century are statistically the same (Test statistic T = 5.064755; Xi2(.05) = 11.1; degrees of freedom = 7).

Attributions of perimortem trauma were made where features of damage to skeletal material were consistent with vital properties of bone fracture as reported by consensus in the literature [38–43]. Such features include: linear fracture propagation with regular, angulated fracture margins (typically obliquely angled to the cortical surface [44]); identical patination between fracture margins and cortical surfaces [44, 45]; smooth and flat planes of cleavage [46–48]; continuation of linear breakage across adjacent elements, internal bevelling of puncture defects and the presence of associated secondary or tertiary fractures.

## Results

The remains from mass graves 101 and 110 represent a minimum of 25 individuals, based on the most frequently occurring identifiable bone element, the petrous part of the left temporal bone. This number is also broadly supported by the comparable numbers of postcranial remains including larger bone elements such as the femora and ulnae, and the smaller elements of the hands and feet (S2 Fig). There is no evidence of any regions of the body being significantly under-represented. Only a single complete cranium is present. Whilst much of the assemblage was disarticulated, some bones remained in articulation, indicating soft tissues to have been present at the time they were interred. Given the above points, it is therefore reasonable to conclude that the remains constitute the primary depositions of individuals who were comparatively recently deceased at the time they were deposited, as opposed to a secondary deposition site containing bones previously buried elsewhere such as a charnel deposit.

None of the skeletal elements can be classified as female, while all the remains demonstrate either male morphology or indeterminate sex. In view of the incomplete and fragmentary nature of the material, we would argue that the whole bone sample is predominantly or exclusively male. Two of the individuals were teenagers and the rest were adults. Age at death was estimated to range from mid-teenage to elderly adult (15–66 years of age based on the pubic symphysis [33] and 16–92 based on the auricular surface [34, 35].

There was evidence for the consequences of fire in both mass graves. In burial 110, there was evidence supporting the hypothesis that several attempts at lighting fires inside the pit of burial 110 were made, with several limited ‘lenses’ of cremated material in different areas of the pit. In burial 101, the evidence for burning was very different: typically constituting well-defined and limited areas of marked charring to bones or the ends of articulated sections (such as exposed vertebrae in a spinal section). There was no evidence of ash or in situ burning within the matrix around the bones.

**Fig 4:**
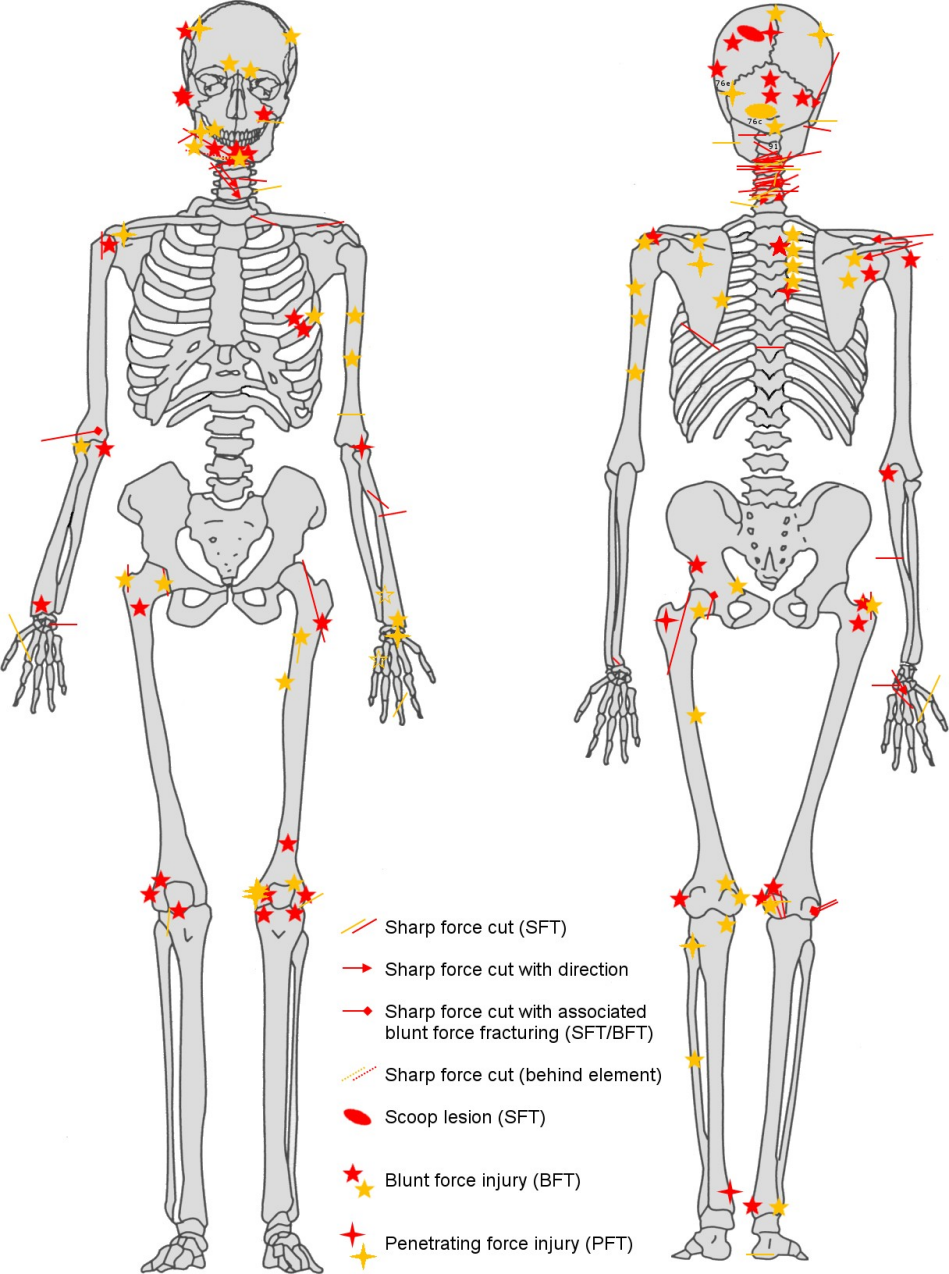
**Distribution of all definite (red) and probable (yellow) perimortem trauma evidenced in the skeletal group deriving from burials 101 and 110 as a whole (image: R. Mikulski).** See also S1 Table for crude prevalences of trauma types by body region.

Perimortem trauma is present on a minimum of 100 bone elements, incorporating sharp force trauma (n = 62 lesions), penetrating force trauma (n = 5) and blunt force trauma (n = 35) (S1 Table). A minimum of nine individuals present evidence of perimortem trauma involving either the cranium or mandible. The postcranial trauma demonstrates a wide distribution throughout the body, although a particular focus is evident across the crania, the cervical region and shoulders (Fig 4). In total there were more wounds present on the back of the skeletons than on the front.

**Fig 5:**
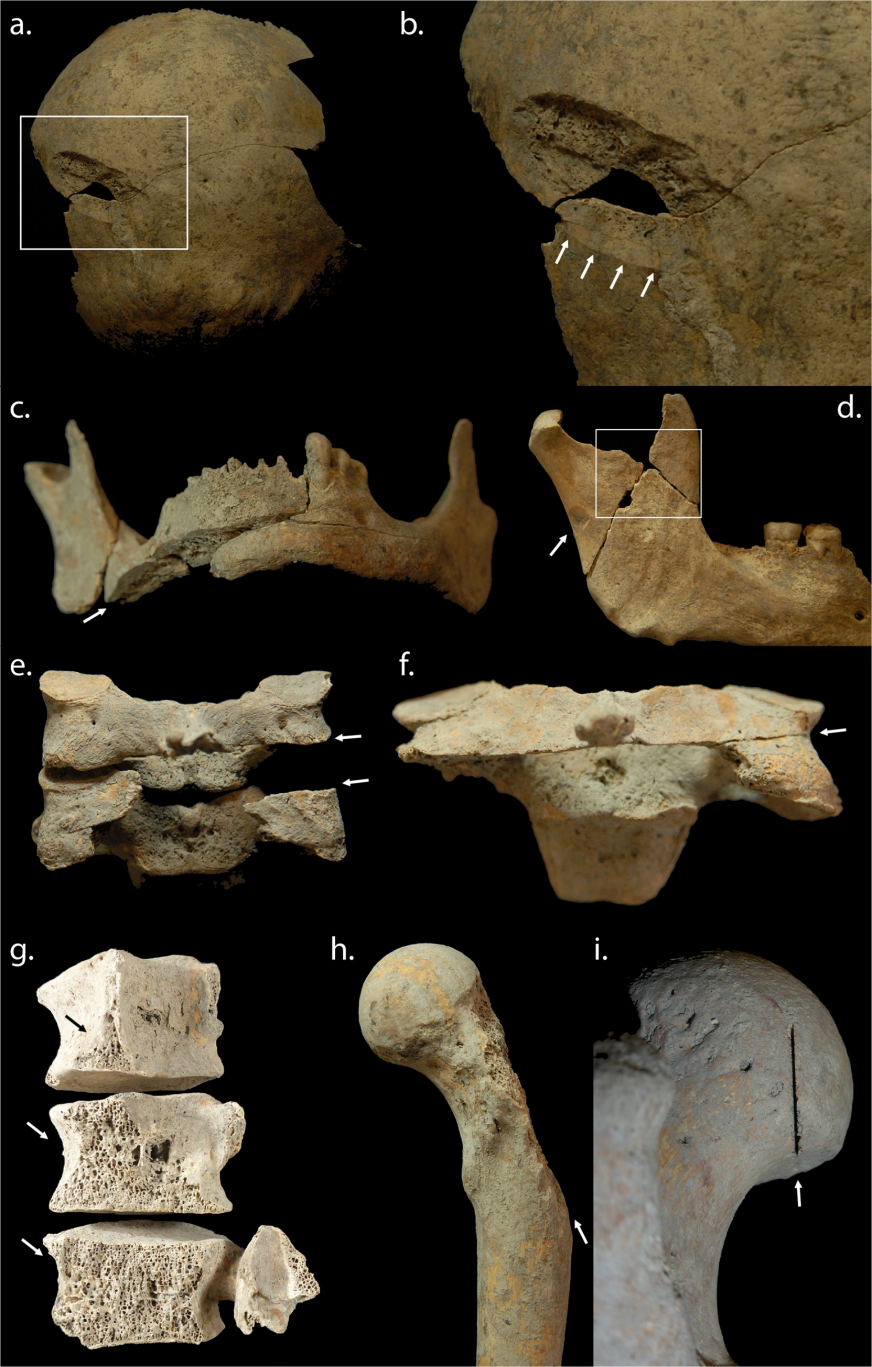
**Examples of sharp force lesions from the Sidon crusader period mass grave deposits**: a) Scoop lesion to posterior left parietal; b) Detail of scoop lesion, showing smooth cut surface of posterior wall indicating direction of cut; c) Smooth cut surface of transected right mandibular body indicating a heavy sharp force blow; d) Well-defined cut to posterior aspect of another right mandible (arrow) with possible penetrating lesion and associated blunt force also evident (box); e) Articulating cervical vertebrae (C4-C5) with evidence of at least two cuts to the posterior right neck; f) Another cervical vertebra (C4) demonstrating a deeply-penetrating cut across the back of the neck that has transected the central and left portions of the inferior neural arch including the left inferior apophyseal facet; g) Articulating lumbar vertebrae (L2-L4) demonstrating associated cut surfaces across the posterior left vertebral body indicating a massively heavy sharp force cut to the posterior lower left side of the torso; h) A left femur demonstrating cut surface with the greater trochanter completely missing; i) A left femur exhibiting a very thin and deeply penetrating linear cut to the posterior aspect of the femoral head, indicative of a different blade category to those evidenced in a)-b), c), g) and h) (photos: R. Mikulski).

**Fig 6:**
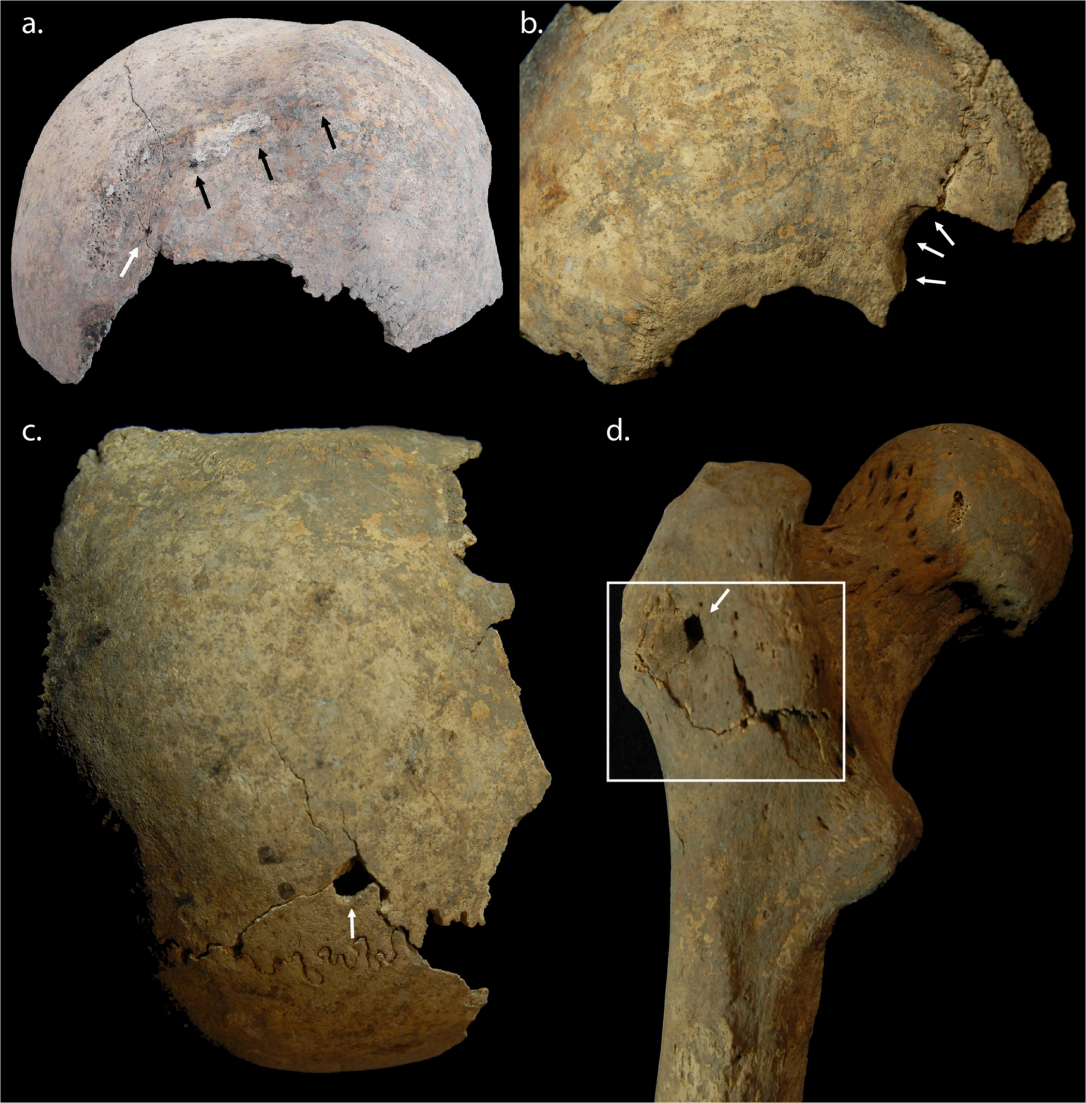
**Examples of penetrating force lesions from the Sidon crusader period mass grave deposits**: a) A group of associated penetrating lesions evidenced at the time of excavation, suggestive of a multiple-pointed implement, such as a spiked mace, rolling across the cranium; b) Large penetrating lesion to the anterior right side of another cranium, demonstrating evidence of healing, indicating this wound was likely inflicted during a separate violent incident; c) Well-defined, rectilinear penetrating lesion to superior left parietal; d) Well-defined rectilinear penetrating lesion to posterior aspect of greater trochanter of a left femur, with associated blunt force evidenced by radiating fractures and an area of crushing to the bone around the inferior aspects of the lesion (photos: R. Mikulski).

Within the assemblage, both light and heavy sharp force trauma is present, as characterised by linear incisions and nicks, scoop lesions indicating a glancing blow, and deeply penetrating cuts with associated blunt force characteristics (Fig 5). The majority of the sharp force lesions are indicative of heavy bladed weapons such as swords and axes. Evidence of penetrating force injuries (also known as puncture injuries) is also present on several crania and a left femur (Fig 6). In two of the crania and the femur, the penetrating lesions are associated with blunt force trauma to the area of bone immediately encompassing the primary lesion, and would be most compatible with a blow from a spiked mace. For one of these crania, this interpretation was arrived at on the basis of multiple punctures arranged in a linear pattern accompanied by signs of blunt force trauma (Fig 6A). The femur presented a similar puncture to the posterior greater trochanter, again associated with a crushing injury (Fig 6D). Some individuals also showed evidence for healed weapon injuries, indicating they survived wounds from past conflicts (Fig 6B).

## Discussion

The demographic characteristics of the human remains, coupled with the presence of widespread weapon-related perimortem trauma, is strong evidence that those interred in these two mass burial deposits were involved in an episode of armed conflict. There is no indication of the presence of any female individuals or children, and all the remains were of males of fighting age. In medieval Europe childhood ended around the age of 12, with progressive involvement in adult activities from that age [49].

DNA analysis has recently been employed to determine the genetic ancestry of some of those found in these mass graves. Of those individuals studied, at least three had recent European ancestry, three were of local Near Eastern ancestry, and two individuals had mixed ancestry [50]. Such findings are consistent with the make-up of crusader society, as represented in the contemporary historical sources [51]. This compares with isotope analyses of burials from the crusader cemetery at Caesarea on the coast further to the south, which showed that the majority of Frankish individuals there spent their childhoods in Europe [52].

Dating of the mass graves using radiocarbon dating, the coin and other artefacts indicate the burials were deposited during the mid-13^th^ century. There were two major attacks on Sidon during this time, and it is most plausible to suggest these mass graves resulted from one of these. The medieval chronicle ‘*L’Estoire de Eracles’* records how in 1253 Mamluk forces from Damascus attacked Sidon while its walls were being rebuilt by King Louis IX of France. Hundreds of the population were killed when the Mamluks sacked the city, with others taken away as prisoners [20]. The chronicle of Jean de Joinville also recorded how King Louis IX, in the Holy Land while leading the seventh crusade, helped other crusaders collect together the decomposing corpses and bury them: “We discovered that the king himself had undertaken to have the bodies of the Christians that the Saracens had killed (as was described earlier) buried. He had personally carried the bodies, all rotting and stinking, to place them in trenches in the ground, and he never once covered his nose, although others did so” [53]. An illumination from the Hours of Jeanne D’Evreux (Fig 2E), although produced decades later than these events, depicts specific details concerning the burial of the victims in which Louis IX was involved. The chronicle ‘*The Templar of Tyre’* recounts how in August 1260 Sidon was again attacked, this time sacked by Mongol forces under the leadership of Kitbuqa, killing some and taking others captive [21]. Following these events, the city remained under crusader control until it was abandoned in 1291.

**Fig 7:**
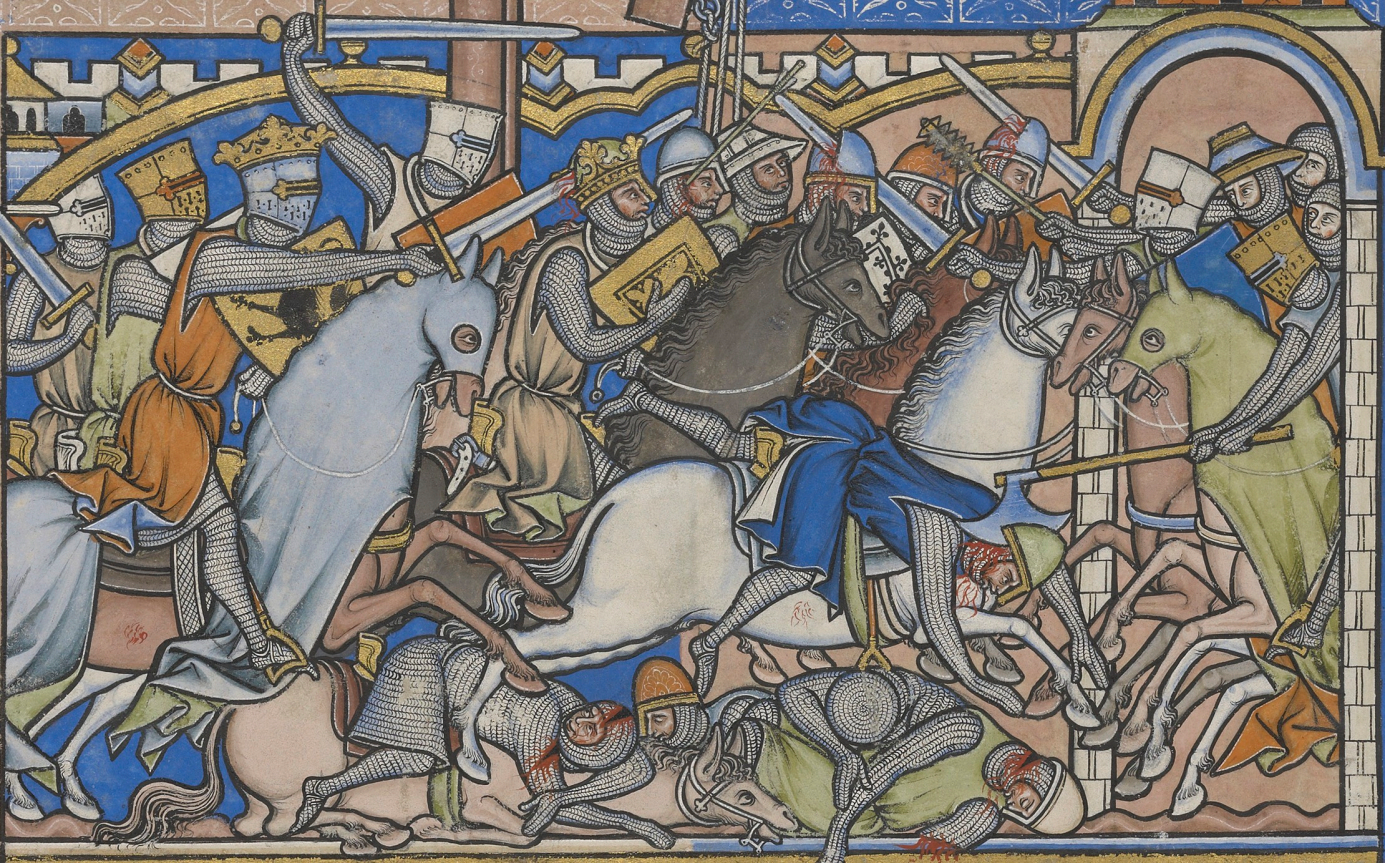
Detail from the Maciejowski Bible, produced in France 1244–1254. Whilst presenting Biblical scenes, these are depicted in the style, social context and material culture of 13th century France and so are a useful source regarding battle injuries and the weapons used by French troops at the time of the raid on Sidon. (Image: The Morgan Library & Museum. MS M.638, fol. 23v. Purchased by J.P. Morgan (1867–1943) in 1916).

**Fig 8:**
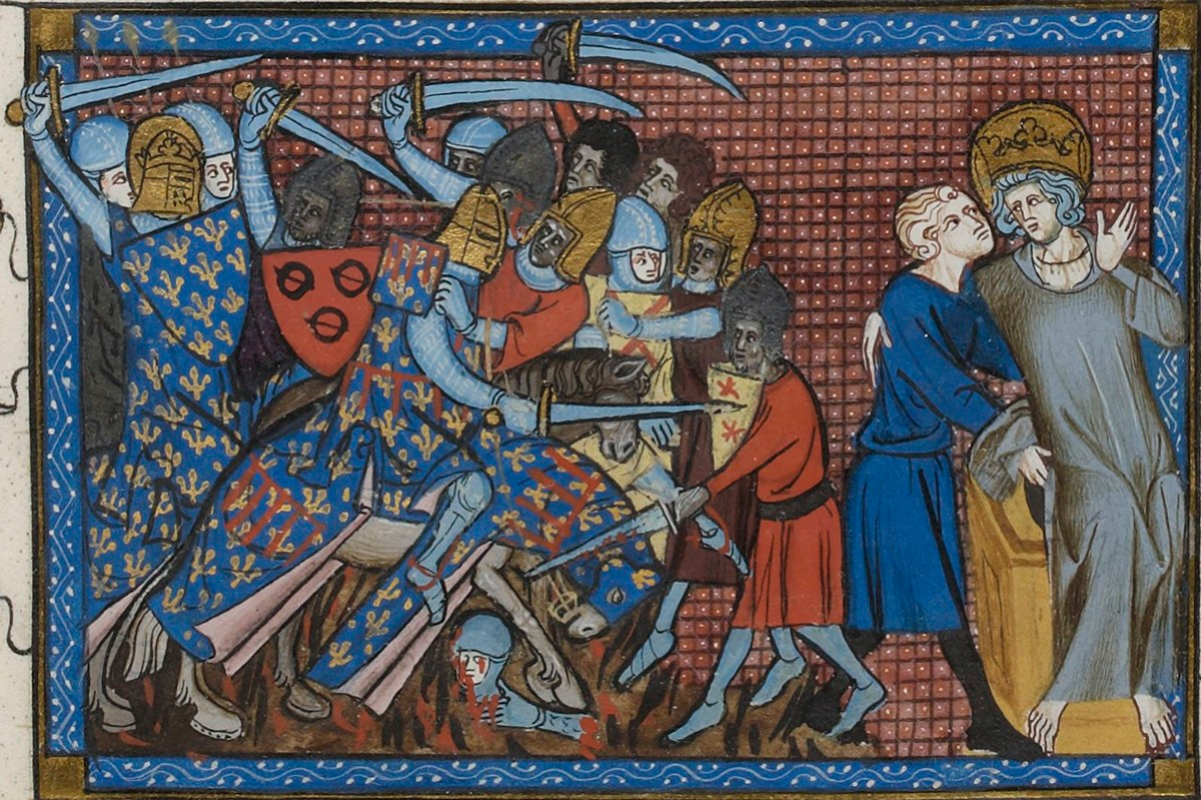
The battle of al Mansourah (1250), as depicted in Guillaume de Saint-Pathus’, Vie et Miracles de saint Louis, 1330–1350, showing the curved swords of the Islamic troops in contrast to the straight-edged swords of the European crusaders. (Image: Gallica Digital library/Bibliothèque nationale de France. Département des Manuscrits. MS Fr.5716, fol.199).

The evidence for trauma most likely reflects injuries sustained in close combat, in which both pointed and bladed weapons were involved, in addition to crushing injuries from blunt force. The large sharp force injuries such as the ‘scoop’ lesion (Fig 5A and 5B), where a slice of bone is removed by a glancing blow, and the other more deeply penetrating sharp force lesions, demonstrate the use of bladed weapons. Crush injuries would be compatible with projectiles from siege engines, such as catapult stones, and blows from rounded maces. Penetrating wounds of square or diamond shape may have been caused by arrows, lances, or blows from the spiked mace. The lesion pattern is consistent with the weaponry of the period including swords, axes, maces, war hammers, arrows, lances and javelins [54, 55; Figs 7 and 8].

**Fig 9:**
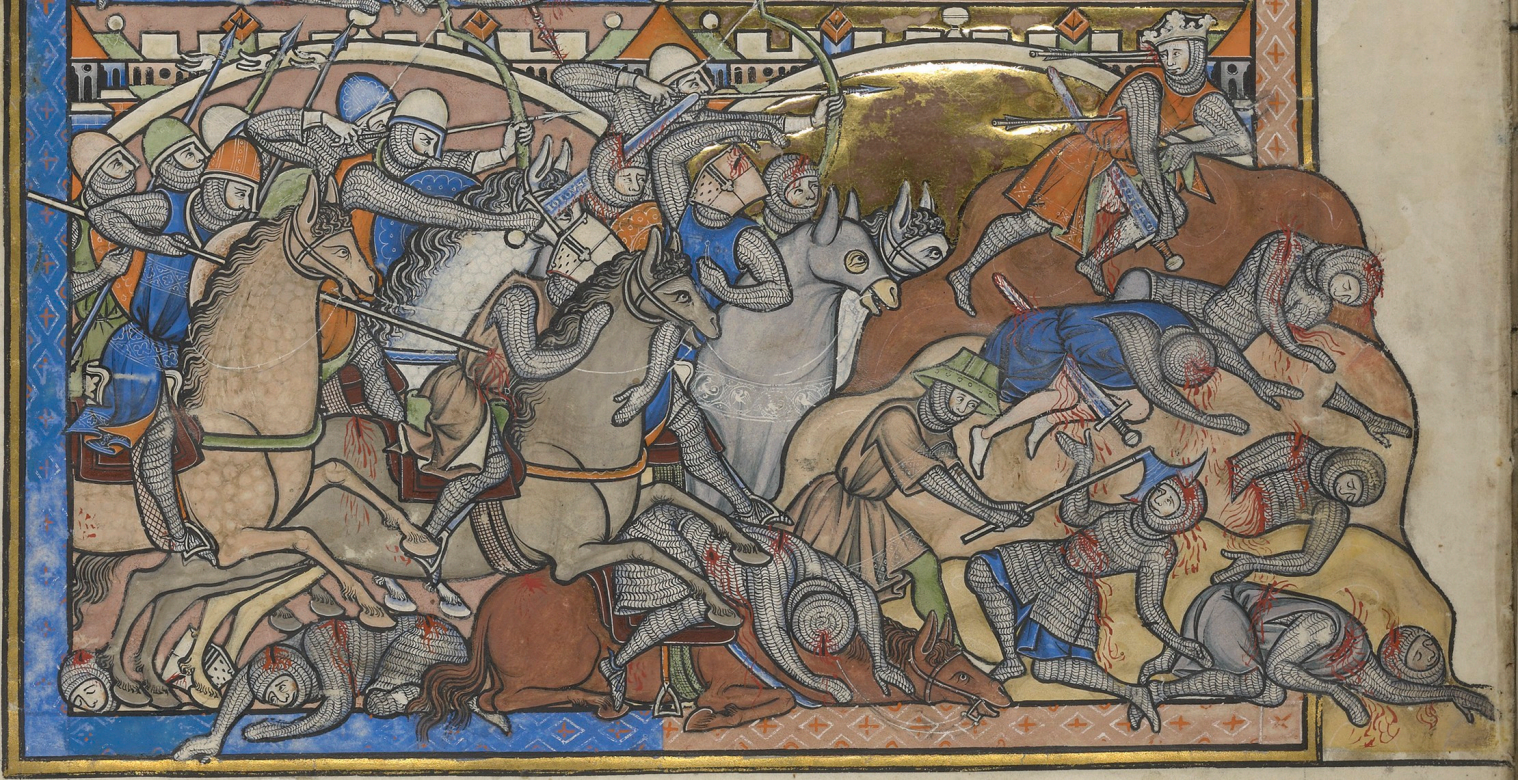
Detail from the Maciejowski Bible, depicting contemporary weapons and the intensity of hand-to-hand combat. (Image: The Morgan Library & Museum. MS M.638, fol. 34v. Purchased by J.P. Morgan (1867–1943) in 1916).

The distribution of lesions in Fig 4 shows many more blows to the head, neck and shoulders than to the lower limbs. This is compatible with both an assault by mounted assailants against footmen [56] and the fighting technique of contemporary near eastern cavalry training [57]. The higher number of wounds to the back of the body, compared with the front, is most likely to indicate that they were wounded while running away from their assailants [58, 59]. A minimum of two individuals exhibited evidence of perimortem sharp force injury to the hands and wrists (with crude prevalence of sharp force injury to all hand elements across the whole group calculated at 40.0%) and these lesions may indicate final attempts to fend off blows once individuals were disarmed. One individual sustained so many wounds (a minimum of 12 injuries involving a minimum of 16 skeletal elements) that it may represent an incident of overkill, where considerably more violent blows were applied than was actually required to overcome or kill them. Although the individual was incomplete, consisting only of the majority of the head, neck, chest and upper arms, the distribution of perimortem trauma demonstrated a focus on the posterior aspects of the head, neck and shoulders. The concentration of transverse blade wounds to the back of the neck within the whole assemblage (crude prevalence = 96.0%, representing a minimum of 24 cuts in total across the group) may indicate execution of some individuals by decapitation following their capture [4]. Contemporary depictions of battle (e.g. Figs 7–9) corroborate the level of violence observed in the skeletal remains; indeed the transected torso and head, towards the bottom right-hand corner of Fig 9, bears a remarkable resemblance to the incomplete individual described above.

The presence of multiple individuals with healed cranial trauma suggests at least part of the burial population had prior experience of violent conflict. Indeed, one cranial fragment exhibited healed and healing cranial trauma indicating multiple violent encounters in this individual’s life history. This makes an interesting comparison with the crusader period burials from Caesarea, where there were examples of healed trauma from accidents but no examples of weapon injuries at all [5]. These healed weapon injuries in the Sidon material would tend to suggest that at least some of the men at Sidon were professional fighters, rather than civilians who had become involved in the conflict.

The general lack of evidence for animal scavenging is intriguing. It is possible the incomplete state of the human remains and their partial articulation may to some degree have resulted from the removal of extremities by larger animals. Such patterned disarticulation is typical of scavenging by canids [60–62]. In such an urban context the most likely candidates would be domestic dogs, although wild canids such as the Arabian wolf (*Canis lupus arabs*) might have been able to access the remains if they were situated outside the city. Equally, any evidence of associated animal scavenging may have been eroded or lost as a result of subsequent taphonomic processes (e.g. the decay and loss of soft tissue evidence); the degree of fragmentation certainly made the full reconstruction of bones impossible in many cases. However, the generally good state of preservation without canid tooth marks argues against the complete taphonomic loss of the majority of any evidence for animal scavenging. Whilst larger mammalian scavengers have been noted to selectively remove body units, such as the upper or lower limbs, such actions do not occur in isolation, but rather as part of a wider pattern of sequential destruction that leaves recognisable signs on various regions of the skeleton. A hypothesis where scavengers only caused damage to bones that were removed, whilst ignoring the remainder, would be inconsistent with observed examples both from modern forensic cases and experiments with wild and captive animals [60–62]. It is therefore more plausible to take the view that the remains were not exposed to significant scavenging activity, either because of their proximity to human settlement or because the interval between death and burial was relatively short, or a combination of both factors.

The relative scarcity of valuable contemporary artefacts (such as weapons or armour) associated with the remains is consistent with the post-battle stripping and looting of the corpses. The presence of disarticulated and articulated remains within a rectilinear pit demonstrates that the remains of the slain were deliberately collected together and buried [63, 64], and that this occurred at a time when the remains were far from fully skeletonized. Charring on some of the bones indicates either that their bodies had been exposed to fire at the time of the attack on the city, or by those undertaking the later reinterment of the rotting bodies.

## Conclusion

These two mass graves at Sidon provide unequivocal evidence of intergroup violence during the period of the crusades. The minimum number of 25 individuals significantly exceeds the scale of the only other known mass burial context reported for the crusader period [9]. As the remains appear to have been of teenage and adult males, this shows a selected group was exposed to a violent death. A high prevalence of perimortem trauma, incorporating sharp force, penetrating force and blunt force, demonstrates the conflict in which these individuals died was extremely violent and destructive. The concentration of wounds to the head and shoulders of some individuals would be compatible with their being on foot when attacked by mounted assailants [56, 57]. As more wounds were present on the back than on the front of these individuals, this would suggest that some were attacked from behind as they tried to flee. The concentration of sharp force trauma to the back of the necks of some individuals may indicate execution by decapitation after their capture.

The nature of the assemblage and taphonomic change on the remains indicates a relatively short period of post-mortem exposure prior to their final deposition at the base of the town fortification ditch. Based on modern forensic observations it can be estimated that an unburied body would take between 50 and 100 days (or 7 to 14 weeks) to skeletonize at the seasonal range of mean temperatures recorded in modern Lebanon between 1985 and 2015 (Jan 13.3°C—Aug 27.78°C, [65, 66]). As the remains were not fully skeletonised at the point of burial, it is reasonable to propose a period measured in weeks depending on the time of year, between death and deposition.

The remains constitute clear evidence for the systematic clearance of bodies in the aftermath of urban conflict in the medieval period. The Sidon mass graves represent a unique find for the crusader period, and corroborate the contemporary account of John of Joinville, an eyewitness to the Seventh Crusade, and his description of how bodies were managed in the weeks following the sack of a city in the mid-13^th^ century.

## Supporting information

S1 Fig **Examples of metal artefacts recovered from burial 110**: a-b) Base silver denaro of Frederick II, c.1245-1250 (Moorhead and Cook, 2011–2012: 404); c) Silver ring with overlapping terminals; d) Cu alloy arrowhead; e) Kidney-shaped buckle with incised hands; f-g) Circular buckles (images courtesy of Dr Claude Doumet-Serhal/DGA).
(TIF)

S2 Fig Summary of MNI represented by individual counts of postcranial bone elements (MNE). (TIF)

S1 Table Total numbers of elements affected by definite perimortem trauma, by region. (TIF)

## References

[1] Nicholson H. The Crusades. Indianapolis, IN: Hackett Publishing Company; 2009.

[2] Riley-Smith J. The Crusades: A History, 3^rd^ edition. London: Bloomsbury Academic; 2014.

[3] Mitchell PD. Medicine in the Crusades: Warfare, Wounds and the Medieval Surgeon. Cambridge: Cambridge University Press; 2004.

[4] Mitchell PD. The torture of military captives during the crusades to the medieval Middle East. In: Christie N, Yazigi M, editors. Noble Ideals and Bloody Realities: Warfare in the Middle Ages, 378–1492. Leiden and Boston: E.J. Brill; 2006. pp. 97–118.

[5] Mitchell PD. Trauma in the crusader period city of Caesarea: a major port in the medieval eastern Mediterranean. Int. J. Osteoarch. 2006; 16: 493–505.

[6] Ashkenazi D, Golan O, Tal O. An archaeometallurgical study of 13th-century arrowheads and bolts from the crusader castle of Arsuf/Arsur. Archaeometry. 2013; 55: 235–257.

[7] Jackson-Tal RE, Tal O. Crusader glass in context: The destruction of Arsur (Apollonia-Arsuf, Israel), April 1265. J. Glass Stud. 2013; 55: 85–100.

[8] Török B, Barkóczy P, Kovács A, Major B, Vágner Z. Arrowheads and chainmail fragments from the crusader Al-Marqab Citadel (Syria): First archeometallurgical approach. Mater. Manuf. Process. 2017; 32: 916–925.

[9] Mitchell PD, Nagar Y, Ellenblum R. Weapon injuries in the 12th century crusader garrison of Vadum Iacob castle, Galilee. Int. J. Osteoarch. 2006; 16: 145–155.

[10] Bennike P, Otto T, Thrane H, Vandkilde H. Rebellion, combat and massacre: A medieval mass grave at Sandbjerg near Næstved in Denmark. In: Otto T, Thrane H, Vandkilde H, editors. Warfare and Society: Archaeological and Social Anthropological Perspectives. Aarhus: Aarhus Universitetsforlag; 2006. pp. 305–318. doi: 10.1385/JMN:30:3:323

[11] Boucherie A, Jørkov MS, Smith M. Wounded to the bone: Digital microscopic analysis of trauma in a medieval mass grave assemblage (Sandbjerget, Denmark, AD 1300–1350). Int. J. Paleopathol. 2017; 19: 66–79. doi: 10.1016/j.ijpp.2017.10.005 29198401

[12] Fiorato V, Boylston A, Knüsel CJ, editors. Blood Red Roses: The Archaeology of a Mass Grave from Towton, A.D. 1461, 2^nd^ revised edition. Oxford: Oxbow books; 2007.

[13] Thordeman B, editor. Armour from the Battle of Wisby, 1361. 2 vols. Stockholm: Viterhets Historie och Antikvitets Akademien; 1939.

[14] Sanlaville P. Étude Géomorphologique de la Région Littorale du Liban. Beyrouth: University of Lebanon; 1977. French.

[15] Richards DS, editor and translator. The Chronicle of Ibn al-Athir for the Crusading Period from al-Kamil fi’l-Ta’rikh. Part 2—The Years 541-589/1146-1193: The Age of Nur al-Din and Saladin. [Crusade texts in translation, 15]. London: Routledge; 2010.

[16] Pringle D. Pilgrimage to Jerusalem and the Holy Land, 1187–1291. [Crusade Texts in translation 23]. Farnham: Ashgate Publishing; 2012.

[17] Edgington SB. Albert of Aachen’s History of the journey to Jerusalem, Vol. 2: Books 7–12. The First Crusade, 1095–1099. [Crusade texts in translation, 25]. Farnham: Ashgate Publishing Ltd.; 2013.

[18] Babcock EA and Kray AC, editors. A History of deeds done beyond the sea: By William Archbishop of Tyre, (2 volumes). Morningside Heights, NY: Columbia University Press; 1943.

[19] Gabrieli F, editor. Arab Historians of the Crusades. Translated by Costello E. J. London: Routledge & Kegan Paul Ltd; 1984.

[20] Shirley J, editor and translator. Crusader Syria in the Thirteenth Century: The Rothelin Continuation of the History of William of Tyre with Part of the Eracles or Acre Text. Aldershot: Ashgate Publishing; 1999.

[21] Crawford PF, editor and translator. The Templar of Tyre: Part II of the ‘Deeds of the Cypriots’. Aldershot: Ashgate Publishing; 2003.

[22] Collins S. Sidon in the time of St. Louis: archaeological discoveries of the 13th century A.D. Arch. Hist. Lebanon. 2012; 34–35: 415–432

[23] Doumet-Serhal C. Sidon: The 2013 and 2014 seasons of excavation, and 16 years on College Site. Berytus. 2016; 56: 1–44.

[24] Adams BJ, Byrd JE, editors. Commingled Human Remains: Methods in recovery, analysis, and identification. Totowa: Humana Press; 2008.

[25] Osterholtz AJ, Baustian KM, Martin DL, editors. Commingled and Disarticulated Human Remains: Working toward improved theory, method, and data. New York: Springer; 2014.

[26] Esri. "Map of Sidon area" [basemap]. Scale Not Given. "World Imagery". December 20, 2016. https://www.arcgis.com/home/item.html?id=10df2279f9684e4a9f6a7f08febac2a9 (February 20, 2017).

[27] Egan G, Pritchard F. Dress Accessories 1150–1450. Woodbridge: Museum of London/Boydell Press; 2002.

[28] Whitehead R. Buckles 1250–1800. Chelmsford: Greenlight Publishing; 1996.

[29] Pringle D. The Red Tower (al-Burj al-Ahmar): Settlement in the Plain of Sharon at the Time of Crusaders and Mamluks AD 1099–1516. London: Council for British Research in the Levant; 1986.

[30] Moorhead S, Cook B. The coins from the excavation at Sidon. Arch. Hist. Lebanon. 2012; 34–35: 399–405.

[31] Buikstra JE, Ubelaker DH, editors. Standards for Data Collection from Human Skeletal Remains. Proceedings of a Seminar at the Field Museum of Natural History. Fayetteville, AR: Arkansas Archaeological Survey; 1994. doi: 10.1073/pnas.91.6.2091

[32] Powers N, editor. The Human osteology method statement. London: Museum of London. Available [online] at: https://www.museumoflondon.org.uk/application/files/4814/5633/5269/osteology-method-statement-revised-2012.pdf

[33] Brooks JM, Suchey ST. Skeletal age determination based on the os pubis: A comparison of the Acsádi-Nemeskéri and Suchey-Brooks methods. Hum. Evol. 1990; 5: 227–238.

[34] Lovejoy CO, Meindl RS, Pryzbeck TR, Mensforth RP. Chronological metamorphosis of the auricular surface of the ilium: a new method for the determination of adult skeletal age at death. Am. J. Phys. Anth. 1985; 68 (1): 15–28 doi: 10.1002/ajpa.1330680103 4061599

[35] Buckberry JL, Chamberlain AT. Age Estimation from the Auricular Surface of the Ilium: A Revised Method. Am. J. Phys. Anth. 2002; 119 (3): 231–239 doi: 10.1002/ajpa.10130 12365035

[36] Schaefer M, Black S, Scheuer L. Juvenile osteology: A laboratory and field manual. London: Academic Press; 2009

[37] Millard AR. Palace Green Library excavations 2013 (PGL13): Chronology of the burials. Durham: Durham University; 2015; June. Technical Report. Available from https://www.dur.ac.uk/resources/archaeology/pdfs/PGL13_Chronology_Report.pdf (accessed: 08/02/2020)

[38] Berryman HE, Symes SA. Recognizing gunshot and blunt cranial trauma through fracture interpretation. In: Reichs KJ, editor. Forensic Osteology: Advances in the Identification of Human Remains, 2^nd^ edition. Springfield, IL: Charles C Thomas; 1998. pp. 333–352.

[39] Passalacqua NV, Fenton TW. Developments in skeletal trauma: blunt-force trauma. In: Dirkmaat DC, editor. A Companion to Forensic Anthropology. London: Wiley-Blackwell; 2015. pp. 400–412.

[40] Symes SA, L’ Abbé EN, Chapman EN, Wolff I, Dirkmaat DC. Interpreting Traumatic Injury to Bone in Medicolegal Investigations. In: Dirkmaat DC, editor. A Companion to Forensic Anthropology. London: Wiley-Blackwell; 2015. pp. 340–389.

[41] Ubelaker DH, Montaperto KM. Trauma Interpretation in the Context of Biological Anthropology. In: Knüsel CJ, Smith MJ, editors. The Routledge Handbook of the Bioarchaeology of Human Conflict London: Routledge; 2013. pp. 25–38.

[42] Wedl VL, Galloway A, editors. Broken Bones: Anthropological Analysis of Blunt Force Trauma Springfield, IL: Charles C. Thomas; 2013.

[43] Wescott DJ. Biomechanics of bone trauma. In: Siegel J, Saukko PJ, Houck M, editors. Encyclopedia of Forensic Sciences. London: Elsevier; 2013. pp. 83–88.

[44] Lovell NC. Trauma analysis in paleopathology. Yearb Phys Anthropol. 1997; 40 (S25): 139–170.

[45] Moraitis K, Spiliopoulou C. Identification and differential diagnosis of perimortem blunt force trauma in tubular long bones. Forensic Sci. Med. Pat. 2006; 4(2): 221–229. doi: 10.1385/FSMP:2:4:221 25868767

[46] Johnson E. Current developments in bone technology. In: Schiffer MB, editor. Advances in archaeological method and theory, vol. 8. Orlando, FL: Academic Press; 1985. pp. 157–235.

[47] Villa P, Mahieu E. Breakage patterns of human long bones. J. Hum. Evol. 1991; 21 (1): 27–48.

[48] Quatrehomme G, İşcan MY. Postmortem skeletal lesions Forensic Sci. Int. 1997; 89 (3): 155–165. 10.1016/s0379-0738(97)00113-8 9363624

[49] Lewis ME. Children of the Golden Minster: St Oswald’s priory and the impact of industrialisation on child health. J. Anthropol. 2013; Article ID 959472.

[50] Haber M, Doumet-Serhal C, Scheib C, Xue Y, Mikulski R, Martiniano R, et al. A transient pulse of genetic admixture from the crusaders in the near east identified from ancient genome sequences. Am. J. Hum. Genet. 2019; 104: 977–984. doi: 10.1016/j.ajhg.2019.03.015 31006515 PMC6506814

[51] Mitchell PD, Millard AR. Approaches to the study of migration during the crusades. Crusades 2013; 12: 1–12.

[52] Mitchell PD, Millard AR. Migration to the medieval Middle East with the crusades. Am. J. Phys. Anthropol. 2009: 140: 518–25. doi: 10.1002/ajpa.21100 19530140

[53] Smith C. Chronicles of the Crusades: Joinville and Villehardouin. London and New York, NY: Penguin; 2008.

[54] Nicolle D. Arms and Armour in the Crusading Era: 1050–1350. 2 vols. White Plains, NY: Kraus International; 1988.

[55] Nicolle D. The reality of Mamluk warfare: weapons, armour and tactics. Al-Masaq. 1994; 7: 77–110.

[56] Łukasik S, Krenz‐Niedbała M, Zdanowicz M, Różański A and Olszacki. Victims of a 17th century massacre in Central Europe: Peri-mortem trauma of castle defenders. Int. J. of Ost., 2019; 29 (2): 281–293

[57] Al-Sarraf S. Close combat weapons of the early Ἁbbāsid Period: Maces, axes and swords. In: Nicolle D, editor, A companion to medieval arms and armour. Woodbridge: Boydell & Brewer; 2002. pp 149–178

[58] Barnes E. The dead do tell tales. In: Williams CK, Bookidis N, editors. Corinth: the Centenary, 1896–1996. Princeton, NJ: American School of Classical Studies at Athens; 2003. pp. 435–443.

[59] Holst MK, Heinemeier J, Hertz E, Jensen P, Løvschal M, Mollerup L, et al. Direct evidence of a large Northern European Roman period martial event and post-battle corpse manipulation. P. Natl. Acad. Sci. USA. 2018; 115: 5920–5925. 10.1073/pnas.1721372115 PMC6003345 29784805

[60] Haglund W. Dogs and coyotes: postmortem involvement with human remains. In: Haglund W and Sorg M, editors. Forensic Taphonomy: the postmortem fate of human remains. New York: CRC; 1997. pp 367–382.

[61] Haglund W, Reay DT, Swindler TR. Canid scavenging/disarticulation sequence of human remains in the Pacific Northwest. J. Forensic Sci. 1989; 34: 587–606. 2738562

[62] Young A, Stillman R, Smith M, Korstjens A. An experimental study of vertebrate scavenging behavior in a northwest European woodland context. J. Forensic Sci. 2014; 59: 1333–42. doi: 10.1111/1556-4029.12468 24611615

[63] Foard G, Curry A. Where are the dead of medieval battles? A preliminary survey. J. Conflict Arch. 2016; 11: 61–77.

[64] Willey P. Prehistoric warfare on the Great Plains: skeletal analysis of the Crow Creek massacre victims. The Evolution of North American Indians series. London: Routledge; 2012

[65] Vass AA. The elusive universal post-mortem interval formula. Forensic Sci. Int. 2011; 204: 34–40. doi: 10.1016/j.forsciint.2010.04.052 20554133

[66] Time and Date AS. Available from: http:timeanddate.com (accessed April 2021).

