# Supplementary figures and images for: Weapon injuries in the crusader mass graves from a 13th century attack on the port city of Sidon (Lebanon)
Source: PLoS One. 2021 Aug 25;16(8):e0256517. doi: 10.1371/journal.pone.0256517 (PMC8386879; doi:10.1371/journal.pone.0256517)

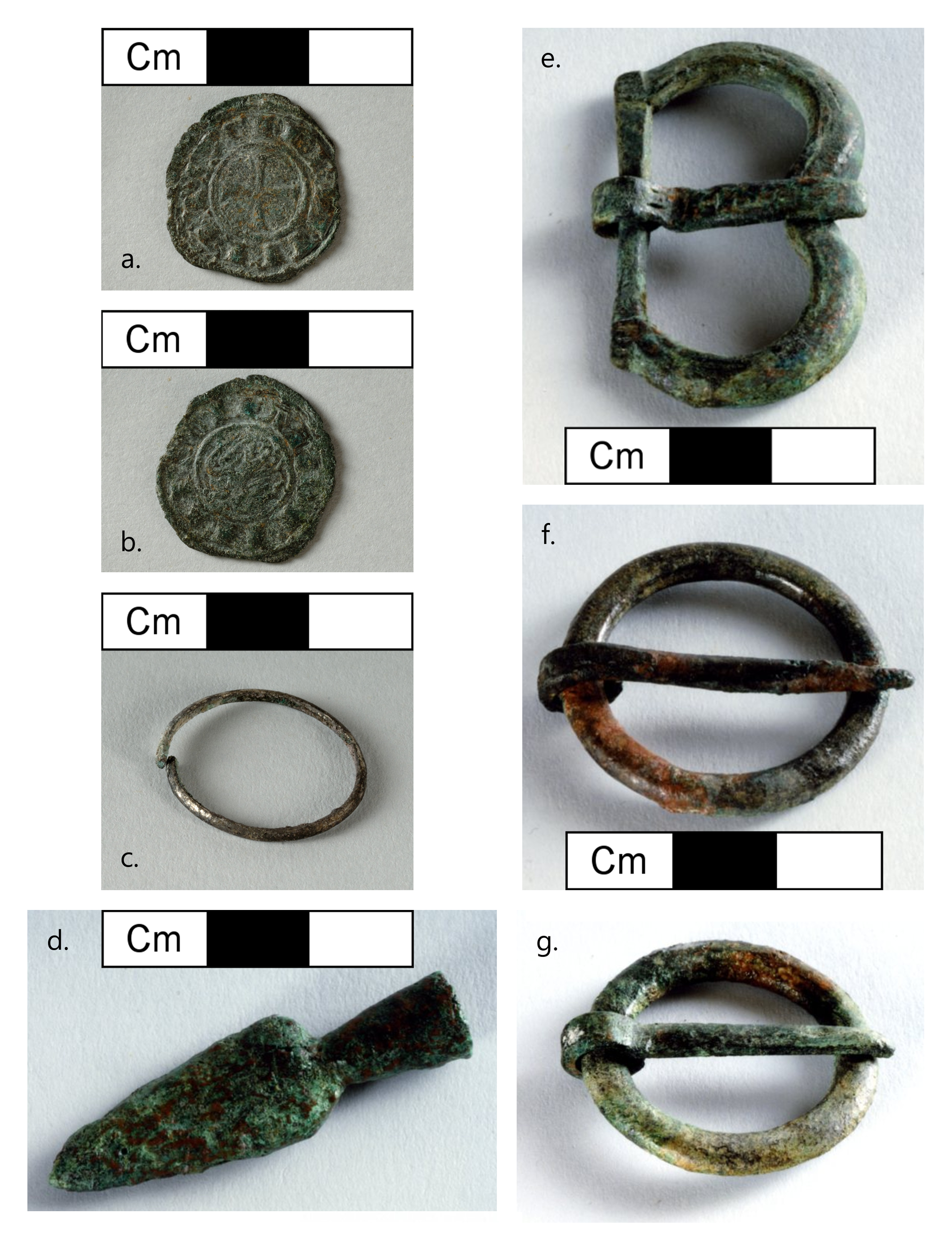

Supplement: S1 Fig — Examples of metal artefacts recovered from burial 110: a-b) Base silver denaro of Frederick II, c.1245-1250 (Moorhead and Cook, 2011–2012: 404); c) Silver ring with overlapping terminals; d) Cu alloy arrowhead; e) Kidney-shaped buckle with incised hands; f-g) Circular buckles (images courtesy of Dr Claude Doumet-Serhal/DGA). (TIF) [file pone.0256517.s001.tif]

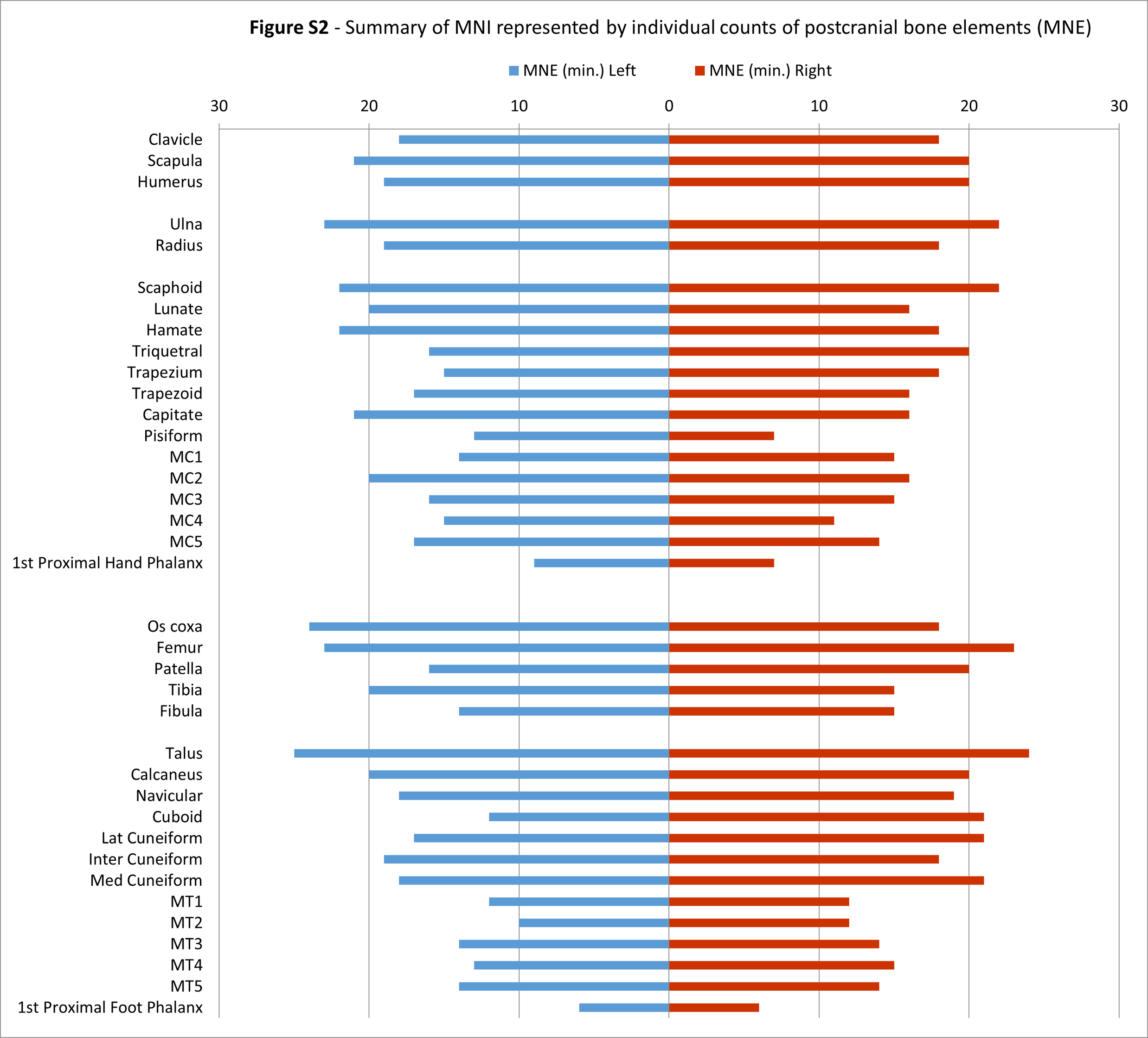

Supplement: S2 Fig — (TIF) [file pone.0256517.s002.tif]

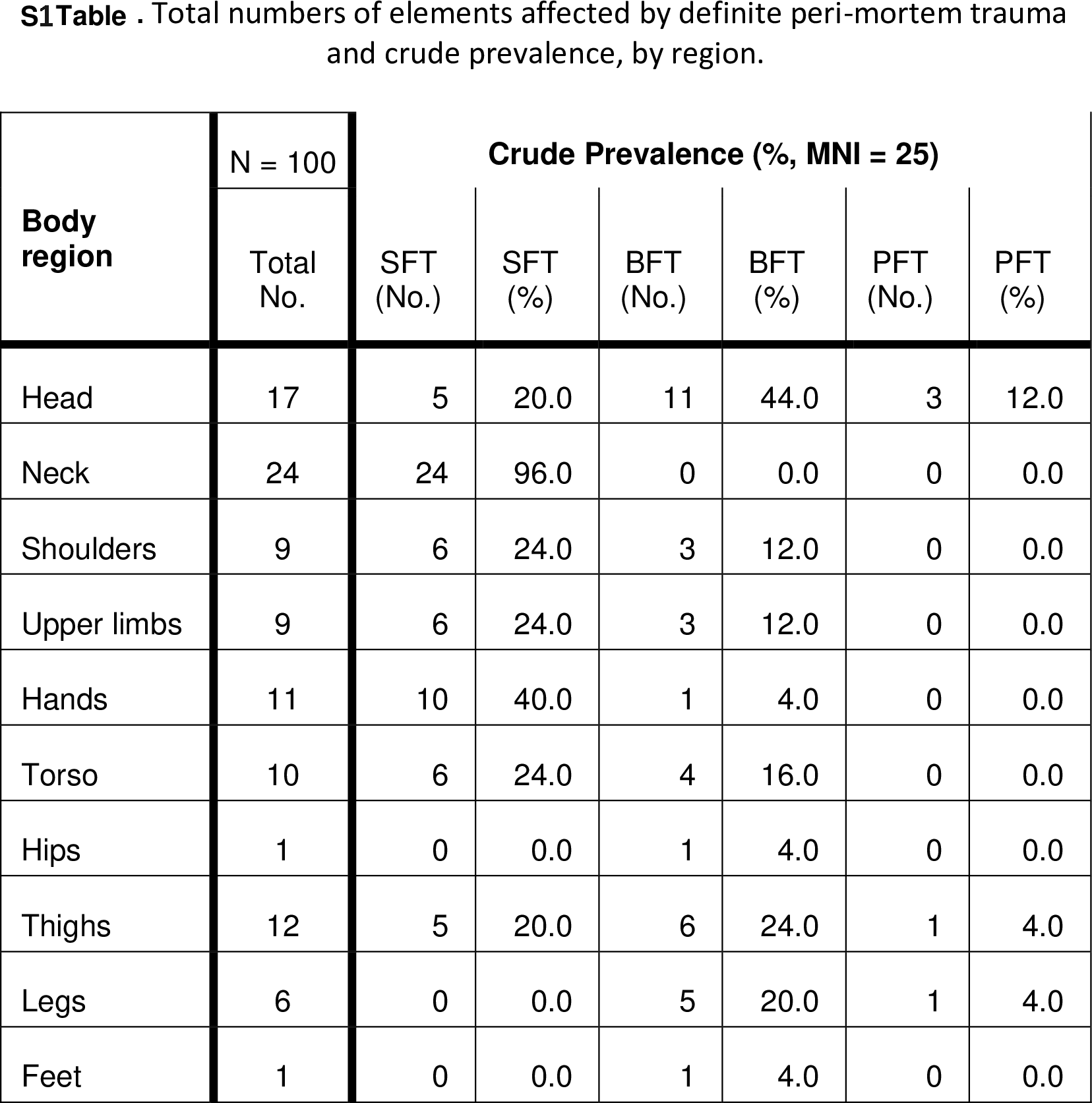

Supplement: S1 Table — (TIF) [file pone.0256517.s003.tif]
